# Bis[tris­(ethane-1,2-diamine)nickel(II)] octa­cyanidomolybdate(IV) penta­hydrate

**DOI:** 10.1107/S1600536809051964

**Published:** 2009-12-09

**Authors:** Qian Jun, Chi Zhang

**Affiliations:** a Molecular Materials Research Center, School of Chemical Engineering, Nanjing University of Science and Technology, 200 Xiaolingwei Road, Nanjing 210094, People’s Republic of China

## Abstract

In the title compound, [Ni(C_2_H_8_N_2_)_3_]_2_[Mo(CN)_8_]·5H_2_O, the Ni^II^ ion is coordinated by six N atoms from three ethane-1,2-diamine ligands in a distorted octa­hedral geometry, while the Mo^IV^ atom is coordianted by eight cyanide ligands. The Ni—N bond distances range from 2.1061 (18) to 2.1425 (18) Å. The Mo—C and C—N distances in the [Mo(CN)_8_] unit range from 2.154 (2) to 2.174 (2) Å and 1.149 (3) to 1.156 (3) Å, respectively. The complex ions and water mol­ecules are linked by weak N—H⋯N/O and O—H⋯N/O hydrogen bonds into a three-demensional structure.

## Related literature

For octacyanidometalates as mol­ecular building units for transition metal complex assemblies, see: Przychodzeń *et al.* (2006 ▶); Withers *et al.* (2005 ▶). For a related structure, see: Liu *et al.* (2008 ▶).
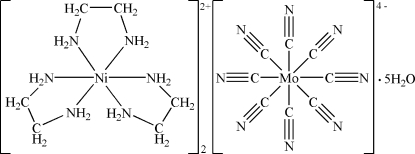


## Experimental

### 

#### Crystal data

[Ni(C_2_H_8_N_2_)_3_]_2_[Mo(CN)_8_]·5H_2_O
*M* *_r_* = 872.18
Monoclinic, 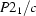

*a* = 13.384 (3) Å
*b* = 16.465 (3) Å
*c* = 21.094 (6) Å
β = 124.45 (2)°
*V* = 3833.2 (18) Å^3^
*Z* = 4
Mo *K*α radiation
μ = 1.35 mm^−1^
*T* = 153 K
0.40 × 0.23 × 0.18 mm

#### Data collection

Rigaku Mercury CCD diffractometer
Absorption correction: multi-scan (*ABSCOR*; Higashi, 1995 ▶) *T*_min_ = 0.770, *T*_max_ = 1.000
36947 measured reflections
6993 independent reflections
6541 reflections with *I* > 2σ(*I*)
*R*_int_ = 0.030

#### Refinement

*R*[*F* ^2^ > 2σ(*F* ^2^)] = 0.028
*wR*(*F* ^2^) = 0.059
*S* = 1.16
6993 reflections
469 parameters
H atoms treated by a mixture of independent and constrained refinement
Δρ_max_ = 0.50 e Å^−3^
Δρ_min_ = −0.47 e Å^−3^

### 

Data collection: *CrystalClear* (Rigaku, 2008) ▶; cell refinement: *CrystalClear*; data reduction: *CrystalClear*; program(s) used to solve structure: *SHELXS97* (Sheldrick, 2008 ▶); program(s) used to refine structure: *SHELXL97* (Sheldrick, 2008 ▶); molecular graphics: *SHELXTL* (Sheldrick, 2008 ▶); software used to prepare material for publication: *SHELXTL*.

## Supplementary Material

Crystal structure: contains datablocks I, global. DOI: 10.1107/S1600536809051964/pv2243sup1.cif

Structure factors: contains datablocks I. DOI: 10.1107/S1600536809051964/pv2243Isup2.hkl

Additional supplementary materials:  crystallographic information; 3D view; checkCIF report

## Figures and Tables

**Table 1:** Hydrogen-bond geometry (Å, °)

*D*—H⋯*A*	*D*—H	H⋯*A*	*D*⋯*A*	*D*—H⋯*A*
N13—H13*B*⋯N8^i^	0.92	2.32	3.138 (3)	148
N11—H11*A*⋯N5^ii^	0.92	2.26	3.149 (3)	163
N11—H11*B*⋯N8^i^	0.92	2.51	3.285 (3)	142
N12—H12*B*⋯O1^ii^	0.92	2.35	3.230 (3)	161
N14—H14*A*⋯N5^i^	0.92	2.35	3.231 (3)	160
N14—H14*B*⋯N4^ii^	0.92	2.40	3.178 (3)	143
N15—H15*A*⋯N7^iii^	0.92	2.38	3.189 (3)	146
N15—H15*B*⋯N4^iv^	0.92	2.34	3.223 (3)	162
N19—H19*B*⋯N4^iv^	0.92	2.40	3.226 (3)	149
N18—H18*B*⋯N1^iii^	0.92	2.48	3.364 (3)	161
N16—H16*B*⋯N1^iii^	0.83 (3)	2.34 (3)	3.141 (3)	162 (2)
N20—H20*A*⋯O5^v^	0.92	2.26	3.152 (3)	164
O3—H3*B*⋯N8^vi^	0.76 (3)	2.32 (3)	3.066 (3)	167 (3)
O4—H4*A*⋯O2^vii^	0.81 (3)	1.99 (3)	2.779 (3)	164 (3)
O4—H4*B*⋯O2^viii^	0.80 (3)	2.15 (3)	2.927 (3)	166 (3)
O5—H5*A*⋯N6^ix^	0.84 (3)	1.93 (3)	2.767 (3)	177 (3)
O5—H5*B*⋯N7^ii^	0.77 (3)	2.04 (3)	2.795 (3)	170 (3)
N13—H13*A*⋯N6	0.92	2.44	3.214 (3)	142
N12—H12*A*⋯N1	0.92	2.23	3.135 (3)	166
N19—H19*A*⋯N2	0.92	2.39	3.207 (3)	149
N17—H17*A*⋯N2	0.92	2.42	3.183 (3)	141
N17—H17*B*⋯N3	0.92	2.50	3.401 (3)	166
N16—H16*A*⋯O1	0.84 (3)	2.35 (3)	3.183 (3)	173 (2)
N20—H20*B*⋯O2	0.92	2.57	3.353 (3)	143
O2—H2*A*⋯N3	0.76 (3)	2.04 (3)	2.788 (3)	169 (3)
O2—H2*B*⋯O5	0.80 (3)	1.89 (3)	2.671 (2)	167 (3)
O1—H1*A*⋯O3	0.73 (3)	2.05 (3)	2.767 (3)	166 (3)
O1—H1*B*⋯N2	0.86 (3)	2.07 (3)	2.906 (3)	161 (3)
O3—H3*A*⋯O4	0.81 (3)	2.01 (3)	2.788 (3)	161 (3)

Symmetry codes: (i) 
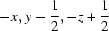
; (ii) 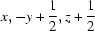; (iii) 
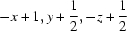
; (iv) 
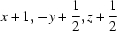
; (v) 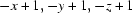; (vi) 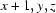; (vii) 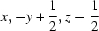; (viii) 
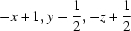
; (ix) 
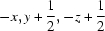
.

**Fig. 1.**
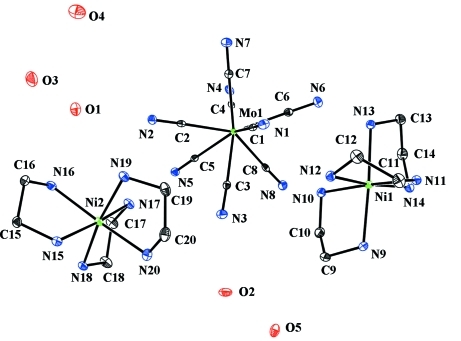
The molecular structure of a portion of the title compound, with atom labels and 30% probability displacement ellipsoids.

**Fig. 2.**
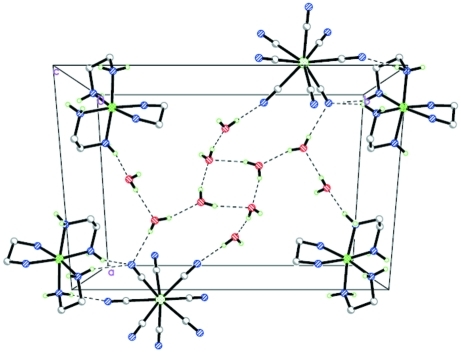
The unit cell packing diagram.

## supplementary crystallographic information

### Comment

Octacyanometalates are well known as molecular building units for transition metal complex-assemblies (Przychodzeń *et al.*, 2006; Withers *et al.*, 2005). The title compound, (I), [Ni(C_2_H_8_N_2_)_3_]_2_[Mo(CN)_8_].5H_2_O, has been prepared in my laboratory which is different from it's analogue [Ni(C_2_H_8_N_2_)_3_]_2_[Mo(CN)_8_].2H_2_O (Liu *et al.*, 2008). The crystal structure of (I) has been presented in this article.

As illustrated in Fig. 1, Ni^2+^ has a octahedral geometry, coordinated by six nitrogen atoms from three ethane-1,2-diamine ligands. The Mo metal center is coordinated by eight cyanide ligands, forming a dodecahedron configuration. The Ni—N bond distances range from 2.1061 (18) to 2.1425 (18) Å. The distances Mo—C and C—N in the [Mo(CN)_8_] unit range from 2.154 (2) to 2.174 (2) and 1.149 (3) to 1.156 (3) Å, respectively.

The complex ions and water molecules are linked by weak N—H···N/O and O—H···N/O hydrogen bonds into a three-demensional structure (Fig. 2).

### Experimental

A solution of K_4_[Mo(CN)_8_].2H_2_O (0.1 mmol) in water (1 ml) was added into a solution of Ni(NO_3_)_2_.6H_2_O (0.4 mmol) and ethylenediamine (0.8 mmol) in water. After vigorous stirring and filtration, 2 ml deionized water and 4 ml (CH_3_)_2_CHOH were successively laid on the surface of above filtrate. Blue block crystals were obtained after five days.

### Refinement

The H atoms bonded to N16 and all the H atoms of water molecules were located in a difference Fourier map and their positional parameters refined with *U*_iso_ = 1.2*U*_eq_, and with the N—H distances are 0.83 (3) Å and 0.84 (3) Å, while the O—H distances range from 0.73 (3) Å to 0.86 (3) Å. Other H atoms were positioned geometrically and refined with riding model, with *U*_iso_ = 1.2*U*_eq_ for H atoms, the N—H bond is 0.92 Å and C—H bond is 0.99 Å in ethane-1,2-diamine.

### Crystal data

**Table:**

[Ni(C_2_H_8_N_2_)_3_]_2_[Mo(CN)_8_]·5H_2_O	*F*(000) = 1824
*M**_r_* = 872.18	*D*_x_ = 1.511 Mg m^−^^3^
Monoclinic, *P*2_1_/*c*	Mo *K*α radiation, λ = 0.71073 Å
Hall symbol: -P 2ybc	Cell parameters from 13627 reflections
*a* = 13.384 (3) Å	θ = 3.1–25.4°
*b* = 16.465 (3) Å	µ = 1.35 mm^−^^1^
*c* = 21.094 (6) Å	*T* = 153 K
β = 124.45 (2)°	Block, blue
*V* = 3833.2 (18) Å^3^	0.40 × 0.23 × 0.18 mm
*Z* = 4	

### Data collection

**Table:**

Rigaku Mercury2 CCD diffractometer	6993 independent reflections
Radiation source: fine-focus sealed tube	6541 reflections with *I* > 2σ(*I*)
graphite	*R*_int_ = 0.030
Detector resolution: 14.6306 pixels mm^-1^	θ_max_ = 25.4°, θ_min_ = 3.1°
dtprofit.ref scans	*h* = −14→16
Absorption correction: multi-scan (*ABSCOR*; Higashi, 1995)	*k* = −19→19
*T*_min_ = 0.770, *T*_max_ = 1.000	*l* = −24→25
36947 measured reflections	

### Refinement

**Table:**

Refinement on *F*^2^	Primary atom site location: structure-invariant direct methods
Least-squares matrix: full	Secondary atom site location: difference Fourier map
*R*[*F*^2^ > 2σ(*F*^2^)] = 0.028	Hydrogen site location: inferred from neighbouring sites
*wR*(*F*^2^) = 0.059	H atoms treated by a mixture of independent and constrained refinement
*S* = 1.16	*w* = 1/[σ^2^(*F*_o_^2^) + (0.0206*P*)^2^ + 2.378*P*] where *P* = (*F*_o_^2^ + 2*F*_c_^2^)/3
6993 reflections	(Δ/σ)_max_ = 0.001
469 parameters	Δρ_max_ = 0.50 e Å^−^^3^
0 restraints	Δρ_min_ = −0.47 e Å^−^^3^

### Special details

**Table:**

Geometry. All e.s.d.'s (except the e.s.d. in the dihedral angle between two l.s. planes) are estimated using the full covariance matrix. The cell e.s.d.'s are taken into account individually in the estimation of e.s.d.'s in distances, angles and torsion angles; correlations between e.s.d.'s in cell parameters are only used when they are defined by crystal symmetry. An approximate (isotropic) treatment of cell e.s.d.'s is used for estimating e.s.d.'s involving l.s. planes.
Refinement. Refinement of *F*^2^ against ALL reflections. The weighted *R*-factor *wR* and goodness of fit *S* are based on *F*^2^, conventional *R*-factors *R* are based on *F*, with *F* set to zero for negative *F*^2^. The threshold expression of *F*^2^ > σ(*F*^2^) is used only for calculating *R*-factors(gt) *etc*. and is not relevant to the choice of reflections for refinement. *R*-factors based on *F*^2^ are statistically about twice as large as those based on *F*, and *R*- factors based on ALL data will be even larger.

### Fractional atomic coordinates and isotropic or equivalent isotropic displacement parameters (Å^2^)

**Table:**

	*x*	*y*	*z*	*U*_iso_*/*U*_eq_	
Ni1	0.10866 (2)	0.093711 (16)	0.382610 (15)	0.01256 (7)	
N9	0.17129 (17)	0.18144 (11)	0.46994 (10)	0.0186 (4)	
H9A	0.1131	0.1910	0.4794	0.022*	
H9B	0.2401	0.1629	0.5146	0.022*	
N13	0.02691 (16)	0.02061 (11)	0.28218 (10)	0.0171 (4)	
H13A	0.0346	0.0454	0.2461	0.020*	
H13B	0.0653	−0.0289	0.2940	0.020*	
N11	0.14481 (16)	−0.00649 (11)	0.45622 (10)	0.0178 (4)	
H11A	0.1255	0.0068	0.4904	0.021*	
H11B	0.0983	−0.0503	0.4276	0.021*	
N10	0.09103 (17)	0.19948 (10)	0.31784 (10)	0.0187 (4)	
H10A	0.1521	0.2007	0.3101	0.022*	
H10B	0.0179	0.1986	0.2707	0.022*	
N12	0.29012 (16)	0.06858 (11)	0.41751 (10)	0.0191 (4)	
H12A	0.2966	0.0743	0.3766	0.023*	
H12B	0.3436	0.1038	0.4558	0.023*	
N14	−0.07363 (16)	0.10052 (11)	0.34935 (11)	0.0198 (4)	
H14A	−0.0871	0.0629	0.3760	0.024*	
H14B	−0.0898	0.1514	0.3594	0.024*	
C13	−0.1028 (2)	0.00881 (14)	0.25067 (13)	0.0230 (5)	
H13C	−0.1126	−0.0392	0.2750	0.028*	
H13D	−0.1488	−0.0010	0.1946	0.028*	
C14	−0.1514 (2)	0.08341 (14)	0.26610 (13)	0.0247 (5)	
H14C	−0.1508	0.1302	0.2369	0.030*	
H14D	−0.2359	0.0739	0.2497	0.030*	
C12	0.3166 (2)	−0.01600 (14)	0.44594 (14)	0.0253 (5)	
H12C	0.4047	−0.0266	0.4746	0.030*	
H12D	0.2739	−0.0544	0.4022	0.030*	
C9	0.1983 (2)	0.25741 (13)	0.44501 (13)	0.0230 (5)	
H9C	0.2772	0.2527	0.4513	0.028*	
H9D	0.2027	0.3036	0.4766	0.028*	
C11	0.2739 (2)	−0.02749 (15)	0.49823 (13)	0.0262 (5)	
H11C	0.2862	−0.0847	0.5157	0.031*	
H11D	0.3216	0.0077	0.5441	0.031*	
C10	0.0983 (2)	0.27144 (14)	0.36156 (13)	0.0228 (5)	
H10C	0.0203	0.2799	0.3557	0.027*	
H10D	0.1160	0.3204	0.3422	0.027*	
Ni2	0.62629 (2)	0.412996 (16)	0.323530 (15)	0.01399 (7)	
N15	0.81820 (15)	0.41301 (10)	0.40039 (10)	0.0166 (4)	
H15A	0.8423	0.4530	0.4368	0.020*	
H15B	0.8442	0.3638	0.4253	0.020*	
N19	0.61894 (16)	0.28343 (11)	0.31654 (10)	0.0189 (4)	
H19A	0.5704	0.2673	0.2660	0.023*	
H19B	0.6954	0.2627	0.3375	0.023*	
N17	0.43848 (16)	0.42928 (11)	0.23603 (11)	0.0214 (4)	
H17A	0.4161	0.3982	0.1937	0.026*	
H17B	0.3924	0.4140	0.2538	0.026*	
N18	0.61559 (15)	0.54090 (10)	0.33354 (10)	0.0177 (4)	
H18A	0.6512	0.5547	0.3843	0.021*	
H18B	0.6562	0.5673	0.3161	0.021*	
N16	0.66946 (17)	0.42220 (13)	0.24089 (11)	0.0190 (4)	
H16A	0.621 (2)	0.3965 (15)	0.2004 (15)	0.023*	
H16B	0.669 (2)	0.4709 (16)	0.2299 (14)	0.023*	
N20	0.59687 (17)	0.38966 (12)	0.41047 (11)	0.0235 (4)	
H20A	0.6479	0.4212	0.4529	0.028*	
H20B	0.5183	0.4023	0.3929	0.028*	
C15	0.87242 (19)	0.42762 (14)	0.35667 (12)	0.0193 (5)	
H15C	0.9547	0.4037	0.3844	0.023*	
H15D	0.8793	0.4867	0.3514	0.023*	
C17	0.4204 (2)	0.51621 (14)	0.21559 (14)	0.0267 (5)	
H17C	0.3329	0.5295	0.1858	0.032*	
H17D	0.4507	0.5288	0.1834	0.032*	
C18	0.4876 (2)	0.56545 (14)	0.28779 (14)	0.0265 (5)	
H18C	0.4807	0.6239	0.2750	0.032*	
H18D	0.4523	0.5566	0.3177	0.032*	
C20	0.6204 (2)	0.30258 (15)	0.43130 (14)	0.0272 (5)	
H20C	0.5820	0.2862	0.4579	0.033*	
H20D	0.7087	0.2930	0.4665	0.033*	
C16	0.7927 (2)	0.38919 (14)	0.27786 (13)	0.0214 (5)	
H16C	0.8244	0.4019	0.2465	0.026*	
H16D	0.7916	0.3294	0.2827	0.026*	
C19	0.5692 (2)	0.25294 (14)	0.35914 (14)	0.0253 (5)	
H19C	0.5911	0.1951	0.3726	0.030*	
H19D	0.4797	0.2573	0.3267	0.030*	
Mo1	0.111354 (15)	0.247118 (10)	0.133637 (10)	0.00983 (6)	
N5	0.13021 (16)	0.43815 (11)	0.09021 (10)	0.0181 (4)	
N4	−0.11016 (17)	0.27440 (11)	−0.05063 (11)	0.0209 (4)	
N3	0.28667 (17)	0.33753 (11)	0.30220 (11)	0.0216 (4)	
N2	0.37242 (16)	0.25891 (11)	0.14992 (10)	0.0184 (4)	
N1	0.27223 (17)	0.10556 (11)	0.26561 (11)	0.0209 (4)	
N8	−0.08227 (17)	0.34005 (11)	0.16017 (11)	0.0221 (4)	
N7	0.11681 (17)	0.09390 (11)	0.03435 (11)	0.0209 (4)	
N6	−0.08414 (18)	0.11512 (12)	0.12231 (11)	0.0252 (4)	
C1	0.21581 (19)	0.15483 (12)	0.22018 (12)	0.0142 (4)	
C2	0.28126 (19)	0.25428 (12)	0.14361 (11)	0.0132 (4)	
C3	0.22444 (18)	0.30556 (12)	0.24402 (12)	0.0141 (4)	
C4	−0.03505 (19)	0.26598 (12)	0.01393 (13)	0.0147 (4)	
C5	0.12387 (18)	0.37181 (13)	0.10493 (11)	0.0130 (4)	
C6	−0.01764 (19)	0.16153 (13)	0.12495 (12)	0.0163 (5)	
C7	0.11469 (18)	0.14779 (13)	0.06841 (12)	0.0145 (4)	
C8	−0.01401 (19)	0.30899 (12)	0.15114 (12)	0.0154 (5)	
O2	0.37621 (16)	0.42633 (12)	0.43668 (10)	0.0305 (4)	
H2A	0.349 (3)	0.3983 (17)	0.4024 (17)	0.037*	
H2B	0.323 (3)	0.4372 (17)	0.4418 (16)	0.037*	
O1	0.47759 (16)	0.34100 (11)	0.07886 (11)	0.0301 (4)	
H1A	0.531 (3)	0.3204 (18)	0.0844 (17)	0.036*	
H1B	0.453 (2)	0.3068 (17)	0.0983 (16)	0.036*	
O3	0.68688 (18)	0.25632 (13)	0.12495 (14)	0.0444 (6)	
H3A	0.675 (3)	0.209 (2)	0.1129 (19)	0.053*	
H3B	0.742 (3)	0.273 (2)	0.1277 (19)	0.053*	
O4	0.59365 (19)	0.09990 (13)	0.07796 (12)	0.0411 (5)	
H4A	0.530 (3)	0.1019 (19)	0.0361 (19)	0.049*	
H4B	0.614 (3)	0.0535 (19)	0.0811 (18)	0.049*	
O5	0.21973 (16)	0.48333 (11)	0.46628 (10)	0.0264 (4)	
H5A	0.178 (2)	0.5220 (17)	0.4379 (16)	0.032*	
H5B	0.184 (2)	0.4617 (17)	0.4796 (16)	0.032*	

### Atomic displacement parameters (Å^2^)

**Table:**

	*U*^11^	*U*^22^	*U*^33^	*U*^12^	*U*^13^	*U*^23^
Ni1	0.01319 (14)	0.01301 (14)	0.01182 (14)	−0.00044 (10)	0.00729 (12)	−0.00008 (10)
N9	0.0217 (10)	0.0201 (10)	0.0164 (10)	−0.0020 (8)	0.0122 (9)	−0.0015 (8)
N13	0.0204 (10)	0.0156 (9)	0.0153 (9)	0.0000 (7)	0.0102 (8)	0.0005 (7)
N11	0.0210 (10)	0.0171 (10)	0.0159 (10)	−0.0031 (7)	0.0108 (8)	−0.0017 (7)
N10	0.0224 (10)	0.0185 (10)	0.0164 (10)	−0.0004 (8)	0.0117 (9)	0.0012 (8)
N12	0.0180 (10)	0.0211 (10)	0.0183 (10)	−0.0017 (8)	0.0103 (8)	−0.0006 (8)
N14	0.0188 (10)	0.0174 (10)	0.0241 (10)	0.0008 (8)	0.0127 (9)	0.0005 (8)
C13	0.0214 (12)	0.0262 (13)	0.0150 (11)	−0.0077 (10)	0.0065 (10)	−0.0025 (10)
C14	0.0144 (11)	0.0316 (14)	0.0217 (12)	−0.0009 (10)	0.0064 (10)	0.0030 (10)
C12	0.0164 (12)	0.0248 (13)	0.0290 (13)	0.0049 (10)	0.0094 (11)	0.0035 (11)
C9	0.0308 (13)	0.0170 (12)	0.0249 (13)	−0.0051 (10)	0.0181 (12)	−0.0059 (10)
C11	0.0229 (13)	0.0250 (13)	0.0220 (13)	0.0028 (10)	0.0076 (11)	0.0084 (10)
C10	0.0311 (13)	0.0163 (12)	0.0280 (13)	0.0038 (10)	0.0209 (12)	0.0030 (10)
Ni2	0.01181 (14)	0.01327 (14)	0.01495 (15)	−0.00075 (10)	0.00641 (12)	−0.00024 (11)
N15	0.0161 (9)	0.0149 (9)	0.0160 (9)	0.0013 (7)	0.0074 (8)	0.0001 (7)
N19	0.0154 (9)	0.0167 (10)	0.0196 (10)	−0.0007 (7)	0.0068 (8)	0.0004 (8)
N17	0.0160 (10)	0.0183 (10)	0.0227 (10)	−0.0025 (8)	0.0065 (9)	−0.0017 (8)
N18	0.0141 (9)	0.0171 (10)	0.0174 (10)	−0.0014 (7)	0.0061 (8)	−0.0026 (8)
N16	0.0199 (10)	0.0182 (10)	0.0146 (10)	−0.0044 (8)	0.0073 (9)	−0.0020 (8)
N20	0.0212 (10)	0.0268 (11)	0.0253 (11)	0.0003 (8)	0.0149 (9)	0.0014 (9)
C15	0.0141 (11)	0.0212 (12)	0.0228 (12)	0.0009 (9)	0.0105 (10)	0.0000 (9)
C17	0.0204 (12)	0.0202 (12)	0.0287 (14)	0.0023 (10)	0.0074 (11)	0.0018 (10)
C18	0.0182 (12)	0.0181 (12)	0.0319 (14)	0.0030 (9)	0.0075 (11)	−0.0004 (10)
C20	0.0254 (13)	0.0310 (14)	0.0269 (13)	0.0036 (11)	0.0159 (12)	0.0099 (11)
C16	0.0201 (12)	0.0235 (12)	0.0246 (12)	−0.0013 (9)	0.0150 (11)	−0.0034 (10)
C19	0.0229 (12)	0.0186 (12)	0.0319 (14)	0.0002 (9)	0.0140 (11)	0.0077 (10)
Mo1	0.01006 (9)	0.00914 (9)	0.00991 (9)	−0.00012 (6)	0.00542 (8)	−0.00013 (7)
N5	0.0196 (10)	0.0171 (10)	0.0177 (10)	0.0000 (8)	0.0106 (9)	0.0000 (8)
N4	0.0184 (10)	0.0190 (10)	0.0172 (11)	−0.0046 (8)	0.0052 (9)	0.0012 (8)
N3	0.0190 (10)	0.0229 (10)	0.0206 (11)	0.0011 (8)	0.0098 (9)	−0.0039 (9)
N2	0.0156 (10)	0.0194 (10)	0.0200 (10)	−0.0009 (7)	0.0099 (9)	−0.0012 (8)
N1	0.0275 (11)	0.0179 (10)	0.0178 (10)	0.0049 (8)	0.0131 (9)	0.0025 (8)
N8	0.0221 (10)	0.0187 (10)	0.0301 (11)	0.0020 (8)	0.0175 (10)	−0.0004 (8)
N7	0.0199 (10)	0.0202 (10)	0.0180 (10)	0.0019 (8)	0.0080 (9)	−0.0012 (8)
N6	0.0288 (11)	0.0201 (11)	0.0305 (12)	−0.0045 (9)	0.0190 (10)	0.0000 (9)
C1	0.0169 (11)	0.0139 (11)	0.0147 (11)	−0.0013 (9)	0.0108 (10)	−0.0035 (9)
C2	0.0166 (11)	0.0101 (10)	0.0110 (10)	0.0006 (8)	0.0066 (9)	−0.0011 (8)
C3	0.0141 (11)	0.0127 (11)	0.0176 (12)	0.0026 (8)	0.0103 (10)	0.0020 (9)
C4	0.0158 (11)	0.0107 (10)	0.0196 (12)	−0.0029 (8)	0.0112 (10)	−0.0011 (9)
C5	0.0091 (10)	0.0179 (12)	0.0101 (10)	−0.0001 (8)	0.0044 (9)	−0.0023 (9)
C6	0.0181 (11)	0.0148 (11)	0.0158 (11)	0.0019 (9)	0.0094 (10)	0.0002 (9)
C7	0.0113 (10)	0.0162 (11)	0.0130 (11)	0.0022 (8)	0.0051 (9)	0.0022 (9)
C8	0.0168 (11)	0.0103 (10)	0.0165 (11)	−0.0030 (8)	0.0080 (10)	0.0009 (8)
O2	0.0196 (9)	0.0414 (11)	0.0261 (10)	−0.0052 (8)	0.0103 (8)	−0.0191 (8)
O1	0.0243 (10)	0.0319 (11)	0.0342 (10)	−0.0006 (8)	0.0166 (9)	0.0035 (8)
O3	0.0301 (11)	0.0394 (12)	0.0691 (15)	−0.0067 (9)	0.0313 (12)	−0.0098 (11)
O4	0.0329 (11)	0.0402 (12)	0.0326 (11)	0.0027 (9)	0.0079 (9)	−0.0032 (10)
O5	0.0286 (10)	0.0261 (10)	0.0318 (10)	0.0075 (7)	0.0214 (9)	0.0102 (8)

### Geometric parameters (Å, °)

**Table:**

Ni1—N9	2.1061 (18)	N17—H17A	0.9200
Ni1—N13	2.1240 (18)	N17—H17B	0.9200
Ni1—N11	2.1253 (18)	N18—C18	1.470 (3)
Ni1—N14	2.1261 (19)	N18—H18A	0.9200
Ni1—N12	2.1422 (19)	N18—H18B	0.9200
Ni1—N10	2.1425 (18)	N16—C16	1.473 (3)
N9—C9	1.479 (3)	N16—H16A	0.84 (3)
N9—H9A	0.9200	N16—H16B	0.83 (3)
N9—H9B	0.9200	N20—C20	1.480 (3)
N13—C13	1.479 (3)	N20—H20A	0.9200
N13—H13A	0.9200	N20—H20B	0.9200
N13—H13B	0.9200	C15—C16	1.515 (3)
N11—C11	1.469 (3)	C15—H15C	0.9900
N11—H11A	0.9200	C15—H15D	0.9900
N11—H11B	0.9200	C17—C18	1.495 (3)
N10—C10	1.470 (3)	C17—H17C	0.9900
N10—H10A	0.9200	C17—H17D	0.9900
N10—H10B	0.9200	C18—H18C	0.9900
N12—C12	1.478 (3)	C18—H18D	0.9900
N12—H12A	0.9200	C20—C19	1.508 (3)
N12—H12B	0.9200	C20—H20C	0.9900
N14—C14	1.476 (3)	C20—H20D	0.9900
N14—H14A	0.9200	C16—H16C	0.9900
N14—H14B	0.9200	C16—H16D	0.9900
C13—C14	1.510 (3)	C19—H19C	0.9900
C13—H13C	0.9900	C19—H19D	0.9900
C13—H13D	0.9900	Mo1—C7	2.154 (2)
C14—H14C	0.9900	Mo1—C6	2.155 (2)
C14—H14D	0.9900	Mo1—C3	2.157 (2)
C12—C11	1.514 (3)	Mo1—C2	2.165 (2)
C12—H12C	0.9900	Mo1—C8	2.166 (2)
C12—H12D	0.9900	Mo1—C1	2.170 (2)
C9—C10	1.509 (3)	Mo1—C4	2.171 (2)
C9—H9C	0.9900	Mo1—C5	2.174 (2)
C9—H9D	0.9900	N5—C5	1.152 (3)
C11—H11C	0.9900	N4—C4	1.156 (3)
C11—H11D	0.9900	N3—C3	1.149 (3)
C10—H10C	0.9900	N2—C2	1.152 (3)
C10—H10D	0.9900	N1—C1	1.152 (3)
Ni2—N20	2.1174 (19)	N8—C8	1.153 (3)
Ni2—N15	2.1248 (19)	N7—C7	1.152 (3)
Ni2—N18	2.1293 (18)	N6—C6	1.150 (3)
Ni2—N16	2.130 (2)	O2—H2A	0.76 (3)
Ni2—N17	2.1326 (19)	O2—H2B	0.80 (3)
Ni2—N19	2.1368 (18)	O1—H1A	0.73 (3)
N15—C15	1.481 (3)	O1—H1B	0.86 (3)
N15—H15A	0.9200	O3—H3A	0.81 (3)
N15—H15B	0.9200	O3—H3B	0.76 (3)
N19—C19	1.476 (3)	O4—H4A	0.81 (3)
N19—H19A	0.9200	O4—H4B	0.80 (3)
N19—H19B	0.9200	O5—H5A	0.84 (3)
N17—C17	1.475 (3)	O5—H5B	0.77 (3)
			
N9—Ni1—N13	170.62 (7)	C19—N19—Ni2	108.45 (13)
N9—Ni1—N11	94.58 (7)	C19—N19—H19A	110.0
N13—Ni1—N11	93.71 (7)	Ni2—N19—H19A	110.0
N9—Ni1—N14	93.85 (7)	C19—N19—H19B	110.0
N13—Ni1—N14	81.56 (7)	Ni2—N19—H19B	110.0
N11—Ni1—N14	91.52 (7)	H19A—N19—H19B	108.4
N9—Ni1—N12	91.09 (7)	C17—N17—Ni2	107.06 (13)
N13—Ni1—N12	94.44 (7)	C17—N17—H17A	110.3
N11—Ni1—N12	81.59 (7)	Ni2—N17—H17A	110.3
N14—Ni1—N12	171.83 (7)	C17—N17—H17B	110.3
N9—Ni1—N10	81.14 (7)	Ni2—N17—H17B	110.3
N13—Ni1—N10	90.91 (7)	H17A—N17—H17B	108.6
N11—Ni1—N10	173.87 (7)	C18—N18—Ni2	109.01 (13)
N14—Ni1—N10	93.14 (7)	C18—N18—H18A	109.9
N12—Ni1—N10	94.05 (7)	Ni2—N18—H18A	109.9
C9—N9—Ni1	108.99 (13)	C18—N18—H18B	109.9
C9—N9—H9A	109.9	Ni2—N18—H18B	109.9
Ni1—N9—H9A	109.9	H18A—N18—H18B	108.3
C9—N9—H9B	109.9	C16—N16—Ni2	106.35 (14)
Ni1—N9—H9B	109.9	C16—N16—H16A	109.9 (17)
H9A—N9—H9B	108.3	Ni2—N16—H16A	112.8 (17)
C13—N13—Ni1	109.53 (13)	C16—N16—H16B	109.2 (17)
C13—N13—H13A	109.8	Ni2—N16—H16B	109.7 (17)
Ni1—N13—H13A	109.8	H16A—N16—H16B	109 (2)
C13—N13—H13B	109.8	C20—N20—Ni2	108.66 (14)
Ni1—N13—H13B	109.8	C20—N20—H20A	110.0
H13A—N13—H13B	108.2	Ni2—N20—H20A	110.0
C11—N11—Ni1	109.11 (13)	C20—N20—H20B	110.0
C11—N11—H11A	109.9	Ni2—N20—H20B	110.0
Ni1—N11—H11A	109.9	H20A—N20—H20B	108.3
C11—N11—H11B	109.9	N15—C15—C16	109.06 (17)
Ni1—N11—H11B	109.9	N15—C15—H15C	109.9
H11A—N11—H11B	108.3	C16—C15—H15C	109.9
C10—N10—Ni1	108.10 (13)	N15—C15—H15D	109.9
C10—N10—H10A	110.1	C16—C15—H15D	109.9
Ni1—N10—H10A	110.1	H15C—C15—H15D	108.3
C10—N10—H10B	110.1	N17—C17—C18	108.89 (19)
Ni1—N10—H10B	110.1	N17—C17—H17C	109.9
H10A—N10—H10B	108.4	C18—C17—H17C	109.9
C12—N12—Ni1	106.64 (13)	N17—C17—H17D	109.9
C12—N12—H12A	110.4	C18—C17—H17D	109.9
Ni1—N12—H12A	110.4	H17C—C17—H17D	108.3
C12—N12—H12B	110.4	N18—C18—C17	109.01 (19)
Ni1—N12—H12B	110.4	N18—C18—H18C	109.9
H12A—N12—H12B	108.6	C17—C18—H18C	109.9
C14—N14—Ni1	106.74 (13)	N18—C18—H18D	109.9
C14—N14—H14A	110.4	C17—C18—H18D	109.9
Ni1—N14—H14A	110.4	H18C—C18—H18D	108.3
C14—N14—H14B	110.4	N20—C20—C19	109.06 (19)
Ni1—N14—H14B	110.4	N20—C20—H20C	109.9
H14A—N14—H14B	108.6	C19—C20—H20C	109.9
N13—C13—C14	109.63 (18)	N20—C20—H20D	109.9
N13—C13—H13C	109.7	C19—C20—H20D	109.9
C14—C13—H13C	109.7	H20C—C20—H20D	108.3
N13—C13—H13D	109.7	N16—C16—C15	108.02 (18)
C14—C13—H13D	109.7	N16—C16—H16C	110.1
H13C—C13—H13D	108.2	C15—C16—H16C	110.1
N14—C14—C13	108.17 (18)	N16—C16—H16D	110.1
N14—C14—H14C	110.1	C15—C16—H16D	110.1
C13—C14—H14C	110.1	H16C—C16—H16D	108.4
N14—C14—H14D	110.1	N19—C19—C20	109.02 (18)
C13—C14—H14D	110.1	N19—C19—H19C	109.9
H14C—C14—H14D	108.4	C20—C19—H19C	109.9
N12—C12—C11	108.12 (18)	N19—C19—H19D	109.9
N12—C12—H12C	110.1	C20—C19—H19D	109.9
C11—C12—H12C	110.1	H19C—C19—H19D	108.3
N12—C12—H12D	110.1	C7—Mo1—C6	75.78 (8)
C11—C12—H12D	110.1	C7—Mo1—C3	140.62 (8)
H12C—C12—H12D	108.4	C6—Mo1—C3	115.01 (8)
N9—C9—C10	108.08 (18)	C7—Mo1—C2	72.74 (7)
N9—C9—H9C	110.1	C6—Mo1—C2	142.27 (8)
C10—C9—H9C	110.1	C3—Mo1—C2	79.27 (8)
N9—C9—H9D	110.1	C7—Mo1—C8	139.83 (8)
C10—C9—H9D	110.1	C6—Mo1—C8	70.20 (8)
H9C—C9—H9D	108.4	C3—Mo1—C8	75.18 (8)
N11—C11—C12	109.32 (18)	C2—Mo1—C8	146.12 (7)
N11—C11—H11C	109.8	C7—Mo1—C1	76.67 (8)
C12—C11—H11C	109.8	C6—Mo1—C1	73.59 (8)
N11—C11—H11D	109.8	C3—Mo1—C1	71.31 (8)
C12—C11—H11D	109.8	C2—Mo1—C1	79.34 (8)
H11C—C11—H11D	108.3	C8—Mo1—C1	112.25 (8)
N10—C10—C9	107.98 (18)	C7—Mo1—C4	72.35 (8)
N10—C10—H10C	110.1	C6—Mo1—C4	80.91 (8)
C9—C10—H10C	110.1	C3—Mo1—C4	144.32 (8)
N10—C10—H10D	110.1	C2—Mo1—C4	108.21 (8)
C9—C10—H10D	110.1	C8—Mo1—C4	81.63 (8)
H10C—C10—H10D	108.4	C1—Mo1—C4	143.77 (8)
N20—Ni2—N15	94.36 (8)	C7—Mo1—C5	120.43 (8)
N20—Ni2—N18	92.16 (7)	C6—Mo1—C5	141.11 (8)
N15—Ni2—N18	92.54 (7)	C3—Mo1—C5	76.22 (8)
N20—Ni2—N16	172.50 (8)	C2—Mo1—C5	74.54 (7)
N15—Ni2—N16	81.60 (8)	C8—Mo1—C5	78.02 (7)
N18—Ni2—N16	94.32 (8)	C1—Mo1—C5	141.38 (8)
N20—Ni2—N17	93.83 (8)	C4—Mo1—C5	72.66 (7)
N15—Ni2—N17	169.95 (7)	N1—C1—Mo1	178.93 (18)
N18—Ni2—N17	81.32 (7)	N2—C2—Mo1	178.90 (19)
N16—Ni2—N17	90.88 (8)	N3—C3—Mo1	178.08 (18)
N20—Ni2—N19	81.64 (7)	N4—C4—Mo1	177.19 (18)
N15—Ni2—N19	92.37 (7)	N5—C5—Mo1	179.32 (18)
N18—Ni2—N19	172.37 (7)	N6—C6—Mo1	178.1 (2)
N16—Ni2—N19	92.17 (7)	N7—C7—Mo1	179.01 (18)
N17—Ni2—N19	94.59 (7)	N8—C8—Mo1	178.22 (17)
C15—N15—Ni2	109.36 (13)	H2A—O2—H2B	106 (3)
C15—N15—H15A	109.8	H1A—O1—H1B	103 (3)
Ni2—N15—H15A	109.8	H3A—O3—H3B	112 (3)
C15—N15—H15B	109.8	H4A—O4—H4B	103 (3)
Ni2—N15—H15B	109.8	H5A—O5—H5B	108 (3)
H15A—N15—H15B	108.2		

### Hydrogen-bond geometry (Å, °)

**Table:**

*D*—H···*A*	*D*—H	H···*A*	*D*···*A*	*D*—H···*A*
N13—H13B···N8^i^	0.92	2.32	3.138 (3)	148
N11—H11A···N5^ii^	0.92	2.26	3.149 (3)	163
N11—H11B···N8^i^	0.92	2.51	3.285 (3)	142
N12—H12B···O1^ii^	0.92	2.35	3.230 (3)	161
N14—H14A···N5^i^	0.92	2.35	3.231 (3)	160
N14—H14B···N4^ii^	0.92	2.40	3.178 (3)	143
N15—H15A···N7^iii^	0.92	2.38	3.189 (3)	146
N15—H15B···N4^iv^	0.92	2.34	3.223 (3)	162
N19—H19B···N4^iv^	0.92	2.40	3.226 (3)	149
N18—H18B···N1^iii^	0.92	2.48	3.364 (3)	161
N16—H16B···N1^iii^	0.83 (3)	2.34 (3)	3.141 (3)	162 (2)
N20—H20A···O5^v^	0.92	2.26	3.152 (3)	164
O3—H3B···N8^vi^	0.76 (3)	2.32 (3)	3.066 (3)	167 (3)
O4—H4A···O2^vii^	0.81 (3)	1.99 (3)	2.779 (3)	164 (3)
O4—H4B···O2^viii^	0.80 (3)	2.15 (3)	2.927 (3)	166 (3)
O5—H5A···N6^ix^	0.84 (3)	1.93 (3)	2.767 (3)	177 (3)
O5—H5B···N7^ii^	0.77 (3)	2.04 (3)	2.795 (3)	170 (3)
N13—H13A···N6	0.92	2.44	3.214 (3)	142
N12—H12A···N1	0.92	2.23	3.135 (3)	166
N19—H19A···N2	0.92	2.39	3.207 (3)	149
N17—H17A···N2	0.92	2.42	3.183 (3)	141
N17—H17B···N3	0.92	2.50	3.401 (3)	166
N16—H16A···O1	0.84 (3)	2.35 (3)	3.183 (3)	173 (2)
N20—H20B···O2	0.92	2.57	3.353 (3)	143
O2—H2A···N3	0.76 (3)	2.04 (3)	2.788 (3)	169 (3)
O2—H2B···O5	0.80 (3)	1.89 (3)	2.671 (2)	167 (3)
O1—H1A···O3	0.73 (3)	2.05 (3)	2.767 (3)	166 (3)
O1—H1B···N2	0.86 (3)	2.07 (3)	2.906 (3)	161 (3)
O3—H3A···O4	0.81 (3)	2.01 (3)	2.788 (3)	161 (3)

Symmetry codes: (i) −*x*, *y*−1/2, −*z*+1/2; (ii) *x*, −*y*+1/2, *z*+1/2; (iii) −*x*+1, *y*+1/2, −*z*+1/2; (iv) *x*+1, −*y*+1/2, *z*+1/2; (v) −*x*+1, −*y*+1, −*z*+1; (vi) *x*+1, *y*, *z*; (vii) *x*, −*y*+1/2, *z*−1/2; (viii) −*x*+1, *y*−1/2, −*z*+1/2; (ix) −*x*, *y*+1/2, −*z*+1/2.
